# Facile synthesis of light harvesting semiconductor bismuth oxychloride nano photo-catalysts for efficient removal of hazardous organic pollutants

**DOI:** 10.1371/journal.pone.0172218

**Published:** 2017-02-28

**Authors:** Zaki S. Seddigi, Mohammed A. Gondal, Umair Baig, Saleh A. Ahmed, M. A. Abdulaziz, Ekram Y. Danish, Mazen M. Khaled, Abul Lais

**Affiliations:** 1 Department of Environmental Health; Faculty of Public Health and Health informatics, Umm Al Qura University, Makkah, Saudi Arabia; 2 Laser Research Group, Department of Physics, King Fahd University of Petroleum & Minerals, Dhahran, Saudi Arabia; 3 Chemistry Department, College of Applied Sciences, Umm Al-Qura University, Makkah, Saudi Arabia; 4 Chemistry Department, Faculty of Science, King Abdulaziz University Jeddah, Saudi Arabia; 5 Department of Chemistry, King Fahd University of Petroleum & Minerals, Dhahran, Saudi Arabia; Institute of Materials Science, GERMANY

## Abstract

In the present work, bismuth oxychloride nanoparticles–a light harvesting semiconductor photocatalyst–were synthesized by a facile hydrolysis route, with sodium bismuthate and hydroxylammonium chloride as the precursor materials. The as-synthesized semiconductor photocatalysts were characterized using X-ray diffraction analysis, Fourier transform infra-red spectroscopy, Raman spectroscopy, Field emission scanning electron microscopy, X-ray photoelectron spectroscopy and Photoluminescence spectroscopy techniques. The crystal structure, morphology, composition, and optical properties of these facile synthesized bismuth oxychloride nanoparticles (BiOCl NPs) were compared to those of traditional bismuth oxychloride. In addition, the photocatalytic performance of facile-synthesized BiOCl NPs and traditional BiOCl, as applied to the removal of hazardous organic dyes under visible light illumination, is thoroughly investigated. Our results reveal that facile-synthesized BiOCl NPs display strong UV-Vis light adsorption, improved charge carrier mobility and an inhibited rate of charge carrier recombination, when compared to traditional BiOCl. These enhancements result in an improved photocatalytic degradation rate of hazardous organic dyes under UV-Vis irradiance. For instance, the facile-synthesized BiOCl NPs attained 100% degradation of methylene blue and methyl orange dyes in approximately 30 mins under UV-Vis irradiation, against 55% degradation for traditional BiOCl under similar experimental conditions.

## Introduction

Water pollution is a colossal problem as it encourages the transmission of waterborne diseases, especially in developing nations. Recently, industries that deal with textile, leather, bleaches and dyes for plastic production, cosmetics, packaging, along with photographic commercial enterprises which utilize coloring materials, have been discovered to release their wastewater into streams without appropriate pre-discharge treatment. These effluents, which contain organic pollutants and dyes that are non-biodegradable and synthetically stable poisons are the main reasons for waterborne infections among human beings [1]. Supplying potable water to the populace has been a pressing need of various governmental and non-governmental organizations. Different techniques have been devised and various others are in process in order to make water suitable for human consumption. Different methods have been applied in treating and removing these contaminants from wastewater like flocculation, adsorption, membrane separation and advanced oxidation process (heterogenous catalysis) [2–10]. Out of the various methodologies and removal methods, development of advanced photo-catalysts remains a “method of choice” for complete degradation of hazardous environmental pollutants (found in drinking water supplies and waste water streams) into harmless carbon dioxide and water [10–20]. Photo-catalysis uses light-sensitive catalysts to accelerate chemical reactions. In brief, a photo-catalyst absorbs photons, which create photo-generated electron–hole charge carriers, and these migrate to the catalyst surface to effect useful chemical transformations, such as dye degradation and solar fuel generation. To achieve its desired outcome, a photo-catalyst must possess certain requisite features: a desirable band gap, appropriate band edge positions (i.e. HOMO & LUMO), large surface area, thermodynamic stability and recyclability [21–22]. There are many classes of compounds that fulfill these criteria, the most prominent of them being metal oxides such as oxides of titanium, zinc, tin etc. As stated earlier, light absorption induces charge separation by photo-exciting electrons from the valence band to the conduction band, forming an electron/hole pair (e-/h+). The positive holes that migrate to the photocatalyst surface have the potential to oxidize harmful organic substrates into harmless substances like CO_2_ and H_2_O [23–24]. For instance, the holes can produce OH radicals by oxidizing OH^**-**^ anions, which can react with pollutants and convert them to more benign substance [25].

Metal oxides (TiO_2_, ZnO, SnO_2_ and CeO_2_) are particularly well-suited to the aforementioned degradation process due to their abundance in nature, biocompatibility and remarkable stability in an array of conditions [26–27]. Metal oxides have a prominent role to play in photocatalytic degradation processes such as the purification of waste water, by killing bacteria and removing other pollutants, which leaves polluted water reusable. Semiconductor photocatalysis has been considered as green, cheap, quick and effective strategy to totally remove the organic pollutants because of its effectiveness, simplicity and low cost [28–29].

In particular, bismuth-based semiconductors have gained tremendous consideration as a result of its strong UV-visible light absorption, lower harmful effects on the environment, and remarkable photocatalytic action against hazardous pollutants [30–31]. Bismuth oxyhalides [BiOX (X = Cl, Br, I)], are an important class of bismuth ternary compounds in view of its optical properties. For quite a long time, they were applied as ferroelectrics, stockpiling materials, and shades. However, contemporary research endeavors have been more inclined towards designing, preparing, and applying bismuth-based semiconductors for photocatalytic uses. The impressive photocatalytic action of bismuth-based semiconductors is to a great extent accounted for by its open crystalline structure and its indirect optical transitions.^28-31^ Owing to the promising results obtained in terms of the photocatalytic properties of the aforementioned bismuth-based semiconductors, it was thought to realize the potential of such materials as effective photocatalyst for complete mineralization of hazardous environmental pollutants into harmless carbon dioxide. Therefore, crystalline, semiconducting bismuth oxychloride nanoparticles were synthesized through a facile, simple hydrolysis route, using sodium bismuthate and hydroxylammonium chloride as the starting materials, and were then characterized by several advanced analytical techniques. The adsorption as well as photocatalytic performance have been appraised by degradation of model organic pollutants (methylene blue dye and methyl orange dye). The results of these findings are presented herein this paper.

## Materials and methods

### Chemical and reagents

All the chemicals and reagents were of analytical grade and used without further purification. Hydroxylammonium chloride (HONH_2_.HCl) and Sodium bismuthate (NaBiO_3_) 80% were procured from Sigma Aldrich, USA. Organic dyes (Methyl orange and Methylene Blue) were procured from Fisher Scientific, New Jersey (USA). Deionized (DI) water was utilized throughout the entire examination, where ever necessary.

### Synthesis of bismuth oxychloride nanocatalyst

In a typical experiment, crystalline bismuth oxychloride nanoparticles (denoted by BiOCl-X, X = 4 and 24) were synthesized through a facile hydrolysis route, with sodium bismuthate and hydroxylammonium chloride as the starting materials. X (X = 4 and 24) denotes the percentage weight of hydroxylammonium chloride. Firstly, 1 g of sodium bismuthate was suspended in 100 ml of deionized water, and stirred by aid of a magnetic stirrer. In the following step, X g (X = 4 and X = 24) of hydroxylammonium chloride was dissolved in 100 ml of DI water and allowed to form a clear solution, then this clear solution was added to the above suspension under magnetic stirring in a dropwise fashion. The stirring was performed for 24 hours under room temperature until a white precipitate was formed. The solid sample was washed with deionized water thoroughly through centrifugation until a neutral pH was obtained. Lastly, the BiOCl formed was harvested and subsequently dried at 70°C for 24 hours in air.

On the other hand, the traditional BiOCl (BiOCl-T) were synthesized via an oxidation-hydrolysis reaction between sodium bismuthate and hydrochloric solution (HCl, 37 wt. %). In a typical synthesis, 4 g of sodium bismuthate was suspended in 50 ml of deionized water under magnetic stirring. In the next step, 20 ml of HCl solution was added into the suspension in a dropwise manner until the sodium bismuthate powder completely dissolved. After adding 250 ml of deionized water to the solution from the previous step, white precipitate of BiOCl-T appeared. This white precipitate of BiOCl-T was collected by centrifugation, and thoroughly washed using deionized water until a neutral pH was obtained. The final product which is traditional BiOCl was then dried at 70°C for 24 hours in air.

### Characterizations

The crystal structure was examined using X-ray diffraction analysis (XRD, Bruker Advance D8) with Cu Kα radiation. The data was collected for the following range: 10° < 2θ < 80°. The morphologies and elemental composition were investigated using field-emission scanning electron microscopy (TESCAN FERA3 FE-SEM) accompanied with an energy-dispersive X-ray spectroscopy (EDS). In other to further affirm the elemental composition, X-ray photoelectron spectroscopic (XPS) analysis were carried out using ESCALAB-250Xi System (Thermos-Scientific), utilizing Al Kα as the incidence radiation source. Attenuated total reflectance Fourier Transform Infrared (ATR-FTIR) spectra were carried out at room temperature using Nicolet iS50 FT-IR Spectrometer. Raman spectroscopy analysis was also examined using Thermo-Scientific Raman analyzer, this was done under ambient condition at room temperature.

### Photo-degradation operation process

#### Sorption performance

Sorption performance study was accomplished to ascertain the sorption kinetics. In a typical sorption study, 50 mg of the as-prepared BiOCl catalysts, were mixed in 100 ml of aqueous solution of MO with a concentration of 20 mg/L; this suspension was kept under dark condition and under magnetic stirring at a speed of 300 rpm and at room temperature for 30 mins. The suspension was then thoroughly centrifuged after each batched experiment, and the absorbance was carried out to calculate the concentration of (Methyl orange dye) MO using UV-Vis spectrophotometer (JASCO V-670). In comparison, this process was repeated for MB (Methylene blue dye) with the same experimental conditions.

#### Photocatalytic activity test

A 500W Xenon lamp (Oriel, USA) was used as light source for the degradation of different compounds on the as-prepared BiOCl. Standard solutions of contaminants (MO and MB dyes) was photodegraded by the addition of a particular amount of the as-prepared catalyst in a typical photo-degradation experiment. Aqueous suspensions of contaminant and catalyst mixture was collected and centrifuged (4000 rpm, 3mins) after irradiation, at fixed intervals to remove particles. The filtrate`s absorbance was then measured by UV-Vis spectroscopy (JASCO V-670) which was in turn used to obtain the concentration of the contaminant. The concentration difference between the initial contaminant solution and the mixture suspension reveals the extent of adsorption of the contaminant by the as-prepared catalyst.

## Results and discussion

### Structural properties

**Fig 1:**
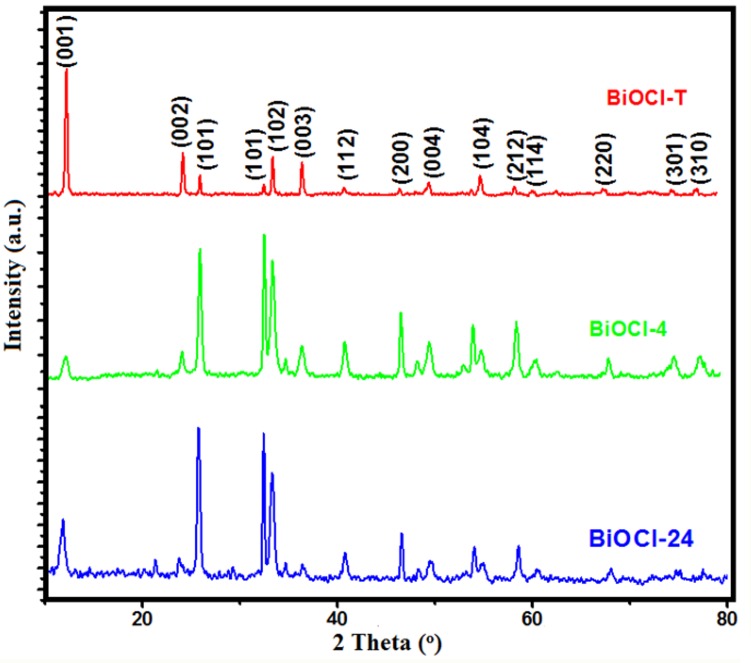
XRD patterns of as-prepared BiOCl-T, BiOCl-4 and BiOCl-24 Photocatalyst.

The crystalline phase of the as-prepared BiOCl-T, BiOCl-4 and BiOCl-24 photo-catalysts is investigated using X-ray diffraction analysis, and the spectra are shown in **Fig 1**. The intense and sharp diffraction peaks (as evidenced by a large height/FWHM ratio) suggest that the as-synthesized BiOCl samples are well-crystallized. All of the diffraction peaks can be indexed to the tetragonal structure of BiOCl (space group: P4/nmm). The (0 0 1) peak at 11.75° is attributed to the periodic stacking structure among [Cl–Bi–O–Bi–O–Cl] layers along the c-axis. The diffraction intensity ratios of I(110)/I(001) were calculated for BiOCl-T, BiOCl-4 and BiOCl-24 and found to be 3.13, 0.26 and 0.62 respectively. This implies that growth of the {001} facets were enhanced in BiOCl-4 and BiOCl-24 as compared to BiOCl-T. In addition, the sizes along the [1 1 0] crystallographic direction exhibited the following order: BiOCl-T > BiOCl-24 > BiOCl-4. From this, it is evident that the crystal growth behavior along the basal ab plane is inhibited in the case of nanosynthesized BiOCl as compared to traditional BiOCl.

### Optical properties

**Fig 2:**
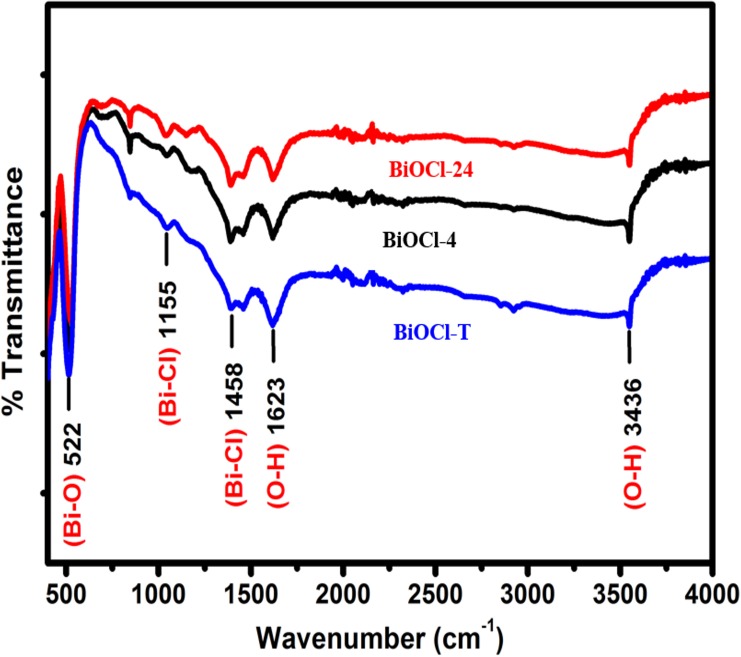
FTIR spectra of as-prepared BiOCl-T, BiOCl-4 and BiOCl-24 Photocatalyst.

The FT-IR spectra of as-prepared BiOCl-T, BiOCl-4 and BiOCl-24 photocatalyst are presented in **Fig 2.** In the FT-IR spectra, the characteristic bands at around 1623 cm^-1^ are attributed to the O-H bending vibrations. The absence of any further prominent bands appearing in the region of 2500 and 1600 cm^**-1**^ reinforces the aforementioned proposition that water molecules are largely absent from all 3 samples, which is indicative of highly pure BiOCl material. The only very strong band occurs at about 528 cm^-1^ in all 3 spectra, and it corresponds to valent symmetrical A_**2u**_-type vibrations of the Bi-O stretching mode, alluding to highly pure BiOCl being formed.

**Fig 3:**
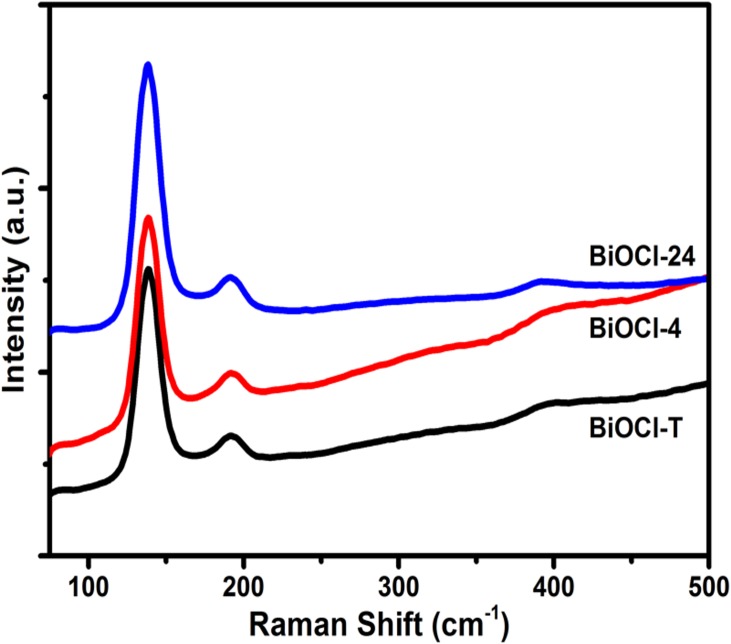
Raman spectra of as-prepared BiOCl-T, BiOCl-4 and BiOCl-24 Photocatalyst.

**Fig 3** shows the Raman spectra of synthesized BiOCl-T, BiOCl-4 and BiOCl-24 photocatalyst. The spectra show one strong and 2 very weak bands. In general, symmetry vibrations give rise to Raman bands of grater intensity than asymmetry vibration. The strong band at 145 cm^-1^ (observed in the nanosynthesized samples BiOCl-T, BiOCl-24 and BiOCl-4) was assigned to A_**1g**_ internal Bi-Cl stretching mode. The weaker band at 201 cm^-1^ can be assigned to the E_**g**_ internal Bi-Cl stretching mode. Lastly, a very weak peak (barely visible) at about 399 cm^-1^ is attributed to E_**g**_ and B_**1g**_ band produced by the motion of oxygen atoms. These wavenumbers are consistent with values reported in the literature, implying that high-purity BiOCl has been synthesized.

**Fig 4:**
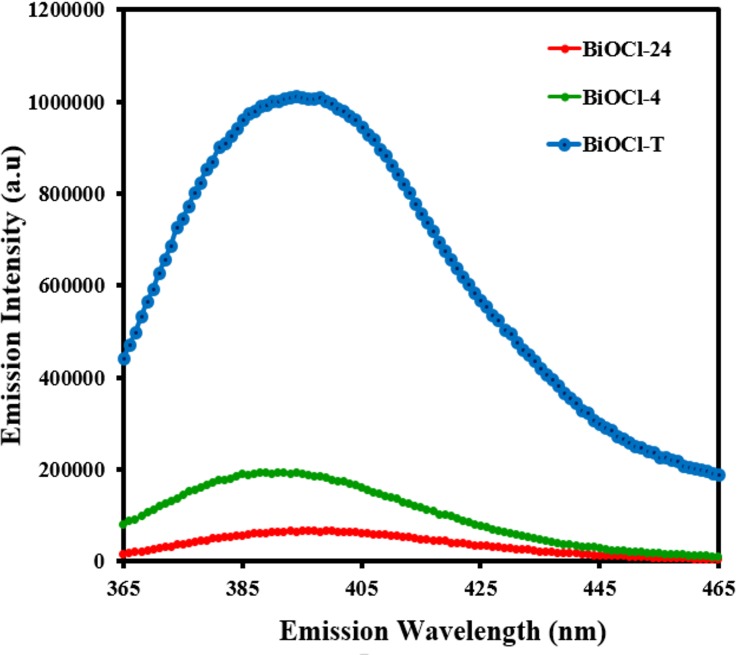
PL spectra of as-prepared BiOCl-T, BiOCl-4 and BiOCl-24 Photocatalyst.

To explore the loss of photogenerated electron-hole pairs by recombination, room temperature photoluminescence spectra of the 3 BiOCl samples (BiOCl-T, BiOCl-4 and BiOCl-24) are recorded and shown in **Fig 4**. The emission spectrum was acquired with an excitation wavelength of 253 nm. There is a strong correlation between the PL emission intensity and charge carrier recombination. A higher PL emission intensity implies a higher recombination of the charge carriers, which adversely affects photocatalytic performance, since the separated charge carriers are primarily responsible for the photocatalytic degradation performance. Hence, a loss of these charge carriers by recombination implies reduced degradation capability of the photocatalyst. The traditional BiOCl (BiOCl-T) displayed a strong emission band centered at 396 nm with high emission intensity. However, the emission intensity of facile synthesized BiOCl-24 and BiOCl-4 were decreased by 80% and 95% respectively, demonstrating the reduction of electron hole pair recombination, which brings about enhanced photocatalytic performance, for the aforementioned reasons. Nano-synthesized BiOCl exhibits lower charge recombination because the migration distance of the charge carriers from the point of generation (within the nanoparticle volume) to the surface is shorter, due to smaller nanoparticle size. This leads to a proportionally shorter migration time, and thus lower volume recombination probability, which leads to more charge carriers migrating to the surface successfully for effecting photocatalytic dye degradation. This results in an enhanced photocatalytic rate.

### Morphological properties

**Fig 5:**
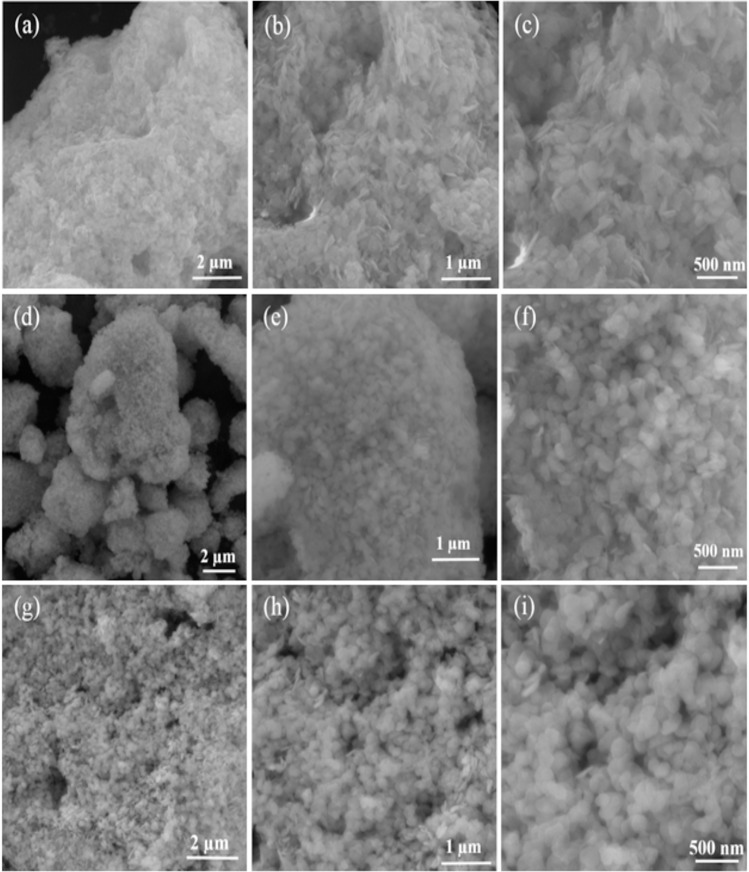
FE-SEM images of BiOCl-T (a-c), BiOCl-4 (d-f) and BiOCl-24 photo-catalyst (g-i) at different magnifications.

The field emission scanning electron (FE-SEM) micrographs of BiOCl-T, BiOCl-4 and BiOCl-24 photocatalyst are shown in **Fig 5**. In **(Fig 5A–5C)**, the morphological topographies of as-prepared BiOCl-T (traditional BiOCl) shows that the BiOCl-T has flake-like structure. However, the morphology of BiOCl-4 and BiOCl-24 nano-synthesized photocatalyst [**Fig 5D–5F and 5G–5I]** are substantially different from that of BiOCl-T. The FE-SEM micrographs in **(Fig 5D–5F and 5G–5I)** clearly prove that the BiOCl-4 and BiOCl-24 are composed of several similarly sized nano-spherical structures assembled hierarchically yielding granules. The granules for different nano-samples (BiOCl-4 and BiOCl-24) have slightly different morphology. Minimization of total system energy drives these nano-spherical structures to coalesce into 3D hierarchical nano structures by self-assembly (obviating the need for any templates) as spherical shapes minimize surface energy. A light-harvesting advantage of the self-assembled nano-structure is that light can be multiply-reflected within the interconnected nano-spheres, which can enhance the light absorption capacity of the photocatalyst. Another advantage of these nanoscale photocatalyst grains is that their specific surface area is larger which leads to an increase in the photocatalysts’ dye adsorption capacity (due to a larger active surface area, since dye molecules are only surface-adsorbed). This eventually leads to an enhanced dye removal rate.

**Fig 6:**
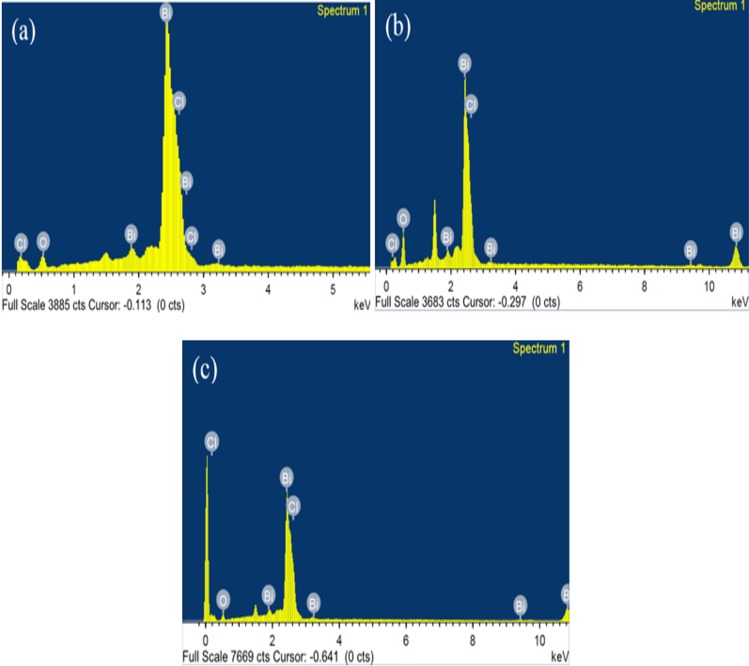
The EDX spectrum of (a) BiOCl-T, (b) BiOCl-4 and (c) BiOCl-24 photo-catalyst.

The elemental composition of BiOCl-T, BiOCl-4 and BiOCl-24 photocatalyst were also confirmed using the EDX Spectra shown in **(Fig 6A, 6B and 6C)** which shows Bi, O, and Cl are present. Quantitative EDX analysis shows that the atomic ratio of Bi/O/Cl in BiOCl-T, BiOCl-4 and BiOCl-24 photocatalyst are 0.97: 0.92: 0.90, 1:0.91:0.92 and 1:0.95:96 respectively, close to 1:1:1, indicating that the composition of the as-synthesized products is indeed BiOCl.

**Fig 7:**
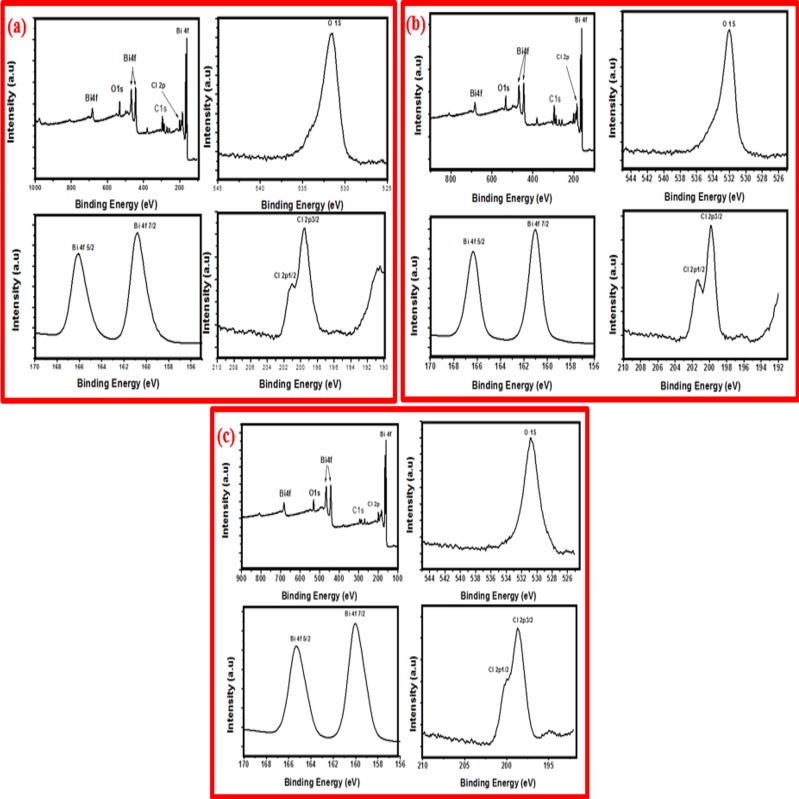
XPS analysis of as-prepared (a) BiOCl-T, (b) BiOCl-4 and (c) BiOCl-24 Photocatalyst.

XPS studies were carried out to further investigate the surface chemical compositions and valence band states of the BiOCl-T, BiOCl-4 and BiOCl-24 photocatalyst, and the spectra are shown in **Fig 7.** The upper left plot in Fig 7A shows a typical survey scan for BiOCl-T over a large energy range at low resolution. This figure demonstrates that BiOCl-T contains Bismuth, Oxygen and Chloride, alluding to the presence of BiOCl photocatalyst. The high resolution scan of Bi 4f, O 1s and Cl 2p states are given in Fig 7A. From high-resolution XPS spectra, a typical 4f7/2 (158.8 eV) and 4f5/2 (164.2 eV) doublet in Bi 4f signal can be found, which is accounted for by the spin-orbit coupling effect, suggesting the tri-valence chemical state in BiOCl sample. The O1s peak at 529.6 eV is attributed to the oxygen anions in the Bi-O bond from the [–O–Bi–O–Bi–O–] slabs in BiOCl. A characteristic doublet for chloride anions was also observed at 2p_**3/2**_ (197.4 eV) and 2p_**1/2**_ (199.0 eV) in Cl 2p, which is accounted for by the characteristic electronic structure of chloride anions.

### Photocatalytic degradation mechanism

**Fig 8:**
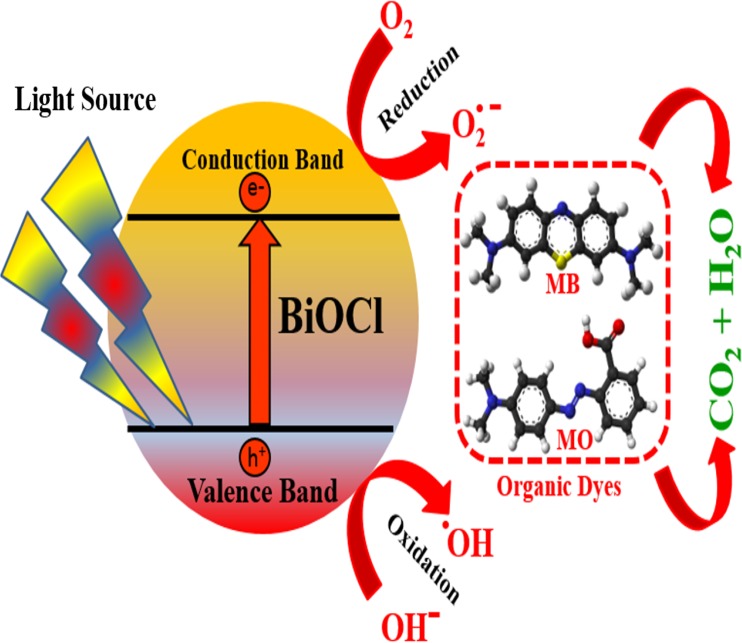
Schematic illustration of the possible photocatalytic reaction mechanism over BiOCl semiconducting nanoparticles.

The scheme in **Fig 8** represents the proposed photocatalytic reaction mechanism over BiOCl semiconducting nanoparticles for the degradation of methyl orange and methylene blue dye. There is photo-generation of electrons (e^-^) and holes (h^+^) by light irradiation upon the photocatalyst in the solution. These e^-^—h^+^ pairs are charge carriers which are used in subsequent redox reaction such as oxygen reduction and water oxidation to yield reactive oxygen species like O_2_^-^, OH^-^. These highly reactive radicals are responsible for oxidative degradation of the pollutant (methylene blue and methyl orange) into mineralized products such as CO_2_ and H_2_O. However, these charge carriers have an intrinsic tendency to recombine (into radiant heat) by volume and surface recombination processes before they have managed to catalyze the desired redox reactions; this reduces the efficiency of the photocatalyst. Hence, there is a strong need to ensure effective separation and migration of the electron and hole pairs.

### Photocatalytic performance

**Fig 9:**
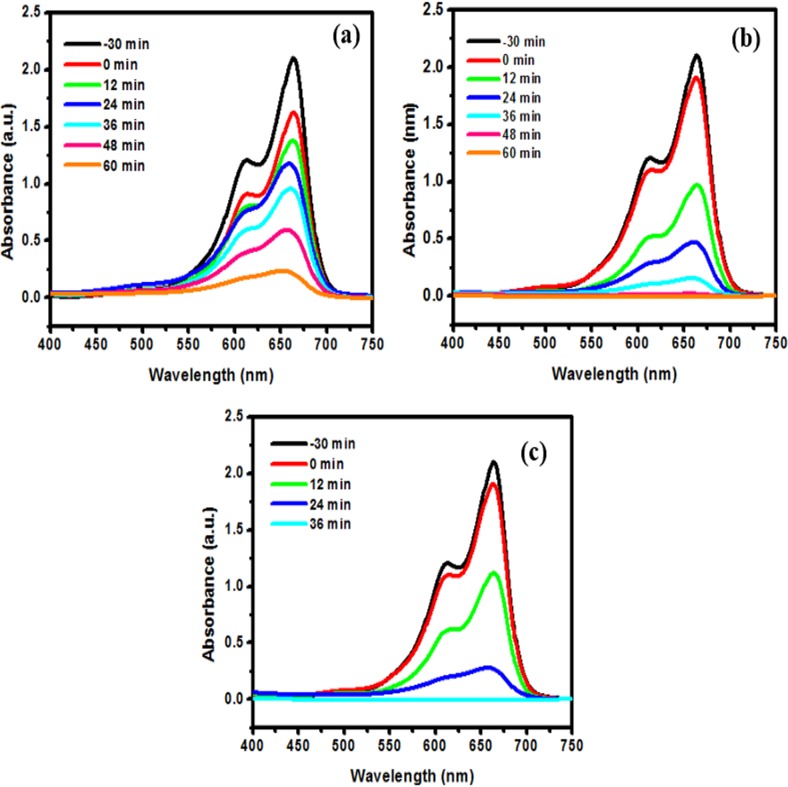
Time resolved UV-Vis spectra of MB solution in the presence of BiOCl-T (a), BiOCl-4(b) and BiOCl-24(c).

**Fig 10:**
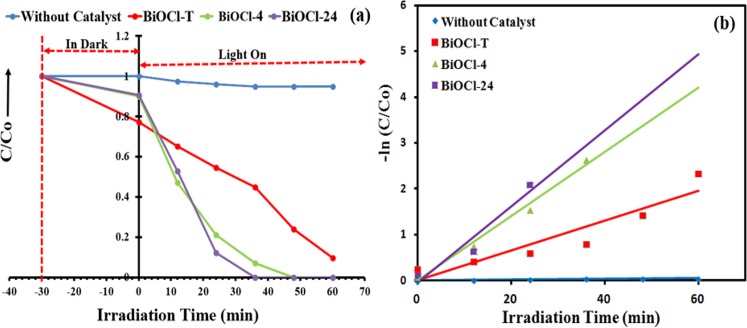
(a) Changes of MB concentration over BiOCl-T, BiOcl-4, BiOCl-24 photocatalystphotocatalystphotocatalysts as a function of irradiation time and photolysis of MB dye (without catalyst) as a function of irradiation time. (b) Pseudo First-order plots for the photocatalytic degradation over BiOCl-T, BiOcl-4, BiOCl-24 catalysts and without catalyst. Experimental conditions: Catalyst = 50 mg, Volume of methylene blue solution = 100 mL, initial methylene blue solution concentration = 10 mg L^-1^.

**Fig 11:**
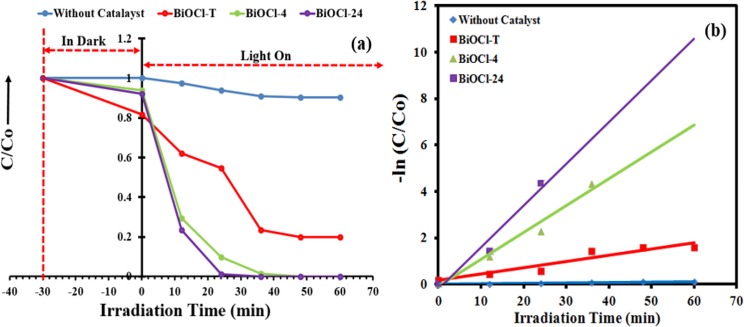
(a) Changes of MO concentration over BiOCl-T, BiOcl-4, BiOCl-24 photocatalystphotocatalystphotocatalysts as a function of irradiation time and photolysis of MB dye (without catalyst) as a function of irradiation time. (b) Pseudo First-order plots for the photocatalytic degradation over BiOCl-T, BiOcl-4, BiOCl-24 catalysts and without catalyst. Experimental conditions: Catalyst = 50 mg, Volume of methyl orange solution = 100 mL, initial methyl orange solution concentration = 10 mg L^-1^.

**Fig 9** depicts temporal changes in the UV-Vis spectra of MB aqueous solution in photo-degradation reaction with the BiOCl-T, BiOCl-4 and BiOCl-24 photocatalyst. The concentration of MB decreased drastically (100%) using BiOCl-24 and BiOCl-4 photocatalyst under UV-visible light irradiation within 36 min and 48 min respectively. However, with BiOCl-T under 60 min of irradiations, the remaining concentrations of MB is still approximately 20%. Displayed in **Fig 10** is the BiOCl-T, BiOCl-4 and BiOCl-24 photocatalytic degradation curves of MB under UV-visible light irradiation source_,_ along with a catalyst-free experimental run (for control purpose). Adsorption-desorption equilibrium was attained between the catalyst particles and the dye solution by continuous stirring in the dark for half an hour before illumination. The photo-catalytic activity was shown by the ratio of the final to initial concentration (C/C_o_) of the dye solution at different time interval upon degradation by the different catalysts under irradiation. Here, C_o_ is the initial concentration of methyl orange after equilibration while C is the concentration at a certain time after irradiation. The activity of the BiOCl-T, BiOCl-4, BiOCl-24 photocatalysts was investigated in the photo-catalytic decomposition of methylene blue dye used as probe molecule under UV-visible light irradiation **(Fig 10).** As shown in Fig 10(A), in the absence of any photocatalyst the MB dye is very stable. It was clear that the facile synthesized BiOCl-4, BiOCl-24 photocatalysts exhibited an enhanced degradation rate and the degradation efficiency was higher than that of traditional BiOCl-T. This is mainly because of enhanced charge carrier transport, improved crystallinity and reduced charge transport resistance. The photo-activity was further investigated by plotting -ln(C/C_o_) against time (Fig 10B), which shows the decomposition kinetics in the form of a linear profile. Hence, using the classical equation: $\ln\left(\frac{C}{Co}\right)=k.t$, where k is the so called degradation rate constant. The k values presented represent a good measurement of the overall photo-degradation rate and are shown in Fig 10(B) by fitting curves of the data for a period of 0–60 min. Similarly, the activity of the BiOCl-T, BiOCl-4 and BiOCl-24 photocatalysts was investigated in the photo-catalytic decomposition of methyl orange dye **(Fig 11).** As shown in **Fig 11(A),** in the absence of any photocatalyst the MO dye is very stable. It is clear that the facile synthesized BiOCl-4, BiOCl-24 photocatalysts exhibited an enhanced degradation rate and the degradation efficiency was higher than that of traditional BiOCl-T. The photo-activity was further investigated by plotting -ln(C/C_o_) against time **(Fig 11B)** showing the decomposition kinetics with linear profile.

The spectroscopic data of MB solution in the presence of BiOCl-24 under visible light irradiation in the UV range indicates the absence of lower hydrocarbons (see S1 File). However for the detection of CO_2_ and other final products of the photo degradation, a control experiment was conducted in the presence of quantitative amounts of oxygen in a closed steel vessel equipped with quartz window. After UV irradiation lasting 30 min in the presence of BiOCl-24 catalyst, the gaseous products were collected and introduced into GC/MS system (Agilent 7890B). The CO_2_ peak was detected at a retention time of 5.79 mins min (see S1 File), which was further analyzed by a mass spectrum to investigate the split pattern. We observed the mass peaks of CO_2_^+^, CO^+^ and O^+^ at 44, 28 and 16 respectively which is assigned to CO_2_ fragments in the mass spectrum (see S1 File). The GC and Mass data shows the complete degradation of the dyes and their conversion into CO_2_ as the major product.

**Fig 12:**
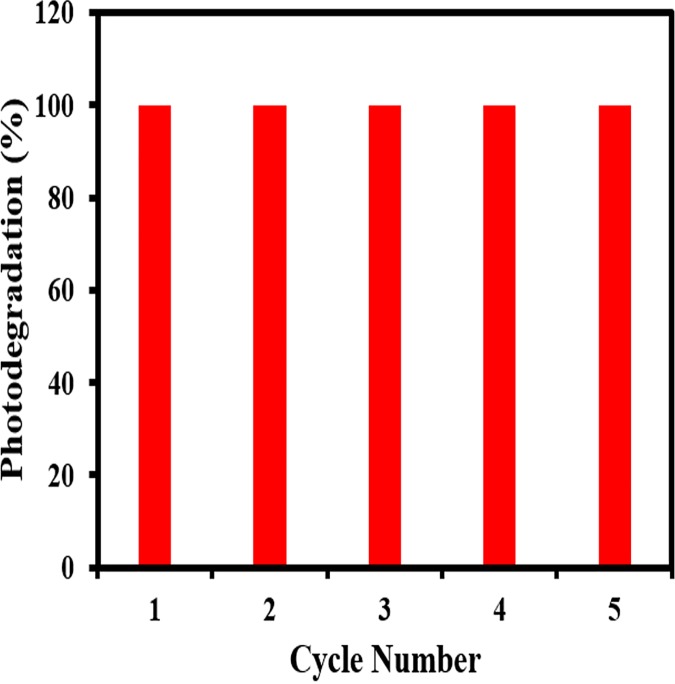
The cycling runs for the photo-degradation of MB dye in the presence of BiOCl-24 under UV-light irradiation.

The photo-corrosion resistance of the photocatalyst is best evaluated by multiple degradation cycles to assess its performance after repeated usage. For this paper, the same photocatalyst sample was used in 5 consecutive runs of degradation experiment, to measure changes in photocatalytic performance under repeated usage. As exemplified in **Fig 12,** the catalyst displays excellent photocatalytic degradation ability even in the 5th usage cycle, attaining almost the same degradation rate after 30 mins for all 5 cycles. This attests to its remarkable photo-corrosion resistance and durability, which is of significant economic value as the catalyst can be recycled for several cycles.

## Conclusions

In the present work, bismuth oxychloride light harvesting semiconductor nanocatalyst was successfully synthesized through a facile hydrolysis route, with sodium bismuthate and hydroxylammonium chloride as the starting materials. The facile synthesized bismuth oxychloride nanoparticles exhibited an enhanced photodegradation performance relative to traditionally synthesized bismuth oxychloride. BiOCl-24 nanocatalyst exhibited the best photo-catalytic activity by degrading methylene blue and methyl orange to 100% under UV-visible radiation in 24 and 36 mins respectively. The improved performance compared to traditional BiOCl was due to enhanced charge carrier transport, improved crystallinity and reduced charge transport resistance. The present work not only provides a new route to obtain the band gap engineered bismuth oxychloride light harvesting semiconductor nanocatalyst, but also allows one to synthesize new photocatalysts having effective applications both under sunlight and artificial light for the degradation and mineralization of hazardous pollutants from drinking water supplies and industrial waste water.

## Supporting information

S1 Fig (a) UV-Vis spectra of MB solution in the presence of BiOCl-24 under visible light irradiation showing that no absorption was noticed for lower hydrocarbon products was noticed, (b) GC chromatogram of CO_2_ showing presence of CO_2_ peak in the degraded product and (c) Mass spectrum of CO_2_.
(TIFF)

S1 File Supporting Information. (DOCX)
