# Supplementary figures and images for: Facile synthesis of light harvesting semiconductor bismuth oxychloride nano photo-catalysts for efficient removal of hazardous organic pollutants
Source: PLoS One. 2017 Feb 28;12(2):e0172218. doi: 10.1371/journal.pone.0172218 (PMC5330479; doi:10.1371/journal.pone.0172218)

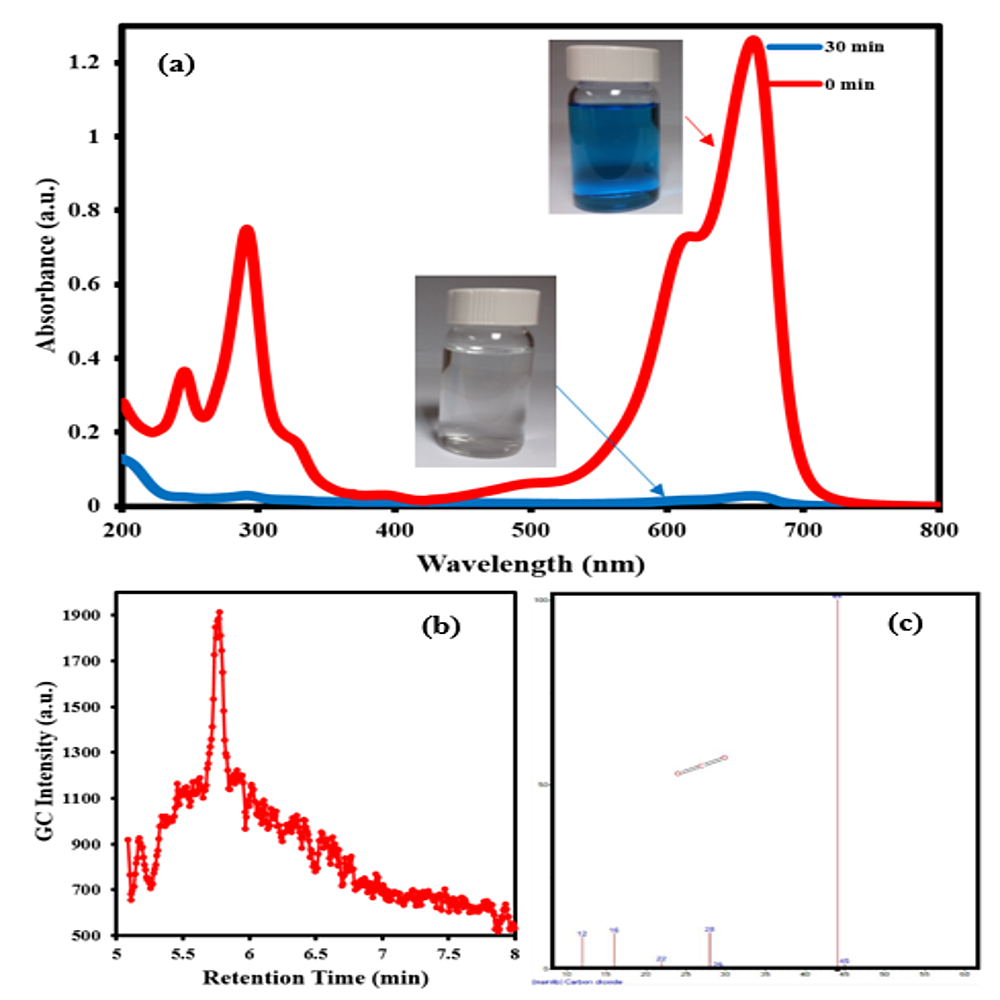

Supplement: S1 Fig — (a) UV-Vis spectra of MB solution in the presence of BiOCl-24 under visible light irradiation showing that no absorption was noticed for lower hydrocarbon products was noticed, (b) GC chromatogram of CO2 showing presence of CO2 peak in the degraded product and (c) Mass spectrum of CO2. (TIFF) [file pone.0172218.s001.tiff]
