# Supplementary material for: Facile synthesis of light harvesting semiconductor bismuth oxychloride nano photo-catalysts for efficient removal of hazardous organic pollutants
Source: PLoS One. 2017 Feb 28;12(2):e0172218. doi: 10.1371/journal.pone.0172218 (PMC5330479; doi:10.1371/journal.pone.0172218)
Supplement: S1 File — (DOCX) [file pone.0172218.s002.docx]

**Supporting Information**

**Facile Synthesis of Light Harvesting Semiconductor Bismuth Oxychloride Nano Photo-catalysts for Efficient Removal of Hazardous Organic Pollutants**

Zaki S. Seddigi^1^, Mohammed A. Gondal^2^*, Umair Baig^2^, Saleh A. Ahmed^3^, M. A. Abdulaziz^1^, Ekram Y. Danish^4^, Mazen M. Khaled^5^ and Abul Lais^2^

^1^Department of Environmental Health; Faculty of Public Health and Health informatics,

Umm Al Qura University, 21955 Makkah, Saudi Arabia

^2^Laser Research Group, Department of Physics, King Fahd University of Petroleum & Minerals,

Dhahran 31261, Saudi Arabia

^3^Chemistry Department, College of Applied Sciences, Umm Al-Qura University, Makkah 21955, Saudi Arabia

^4^Chemistry Department, Faculty of Science, King Abdulaziz University Jeddah, 21589

Saudi Arabia

^5^Department of Chemistry, King Fahd University of Petroleum & Minerals,

Dhahran 31261, Saudi Arabia

***Corresponding authors’ email:** magondal@kfupm.edu.sa (M.A. Gondal)

**Telephone:** +9663-8602351/8603274; **Fax:** +9663-8604281


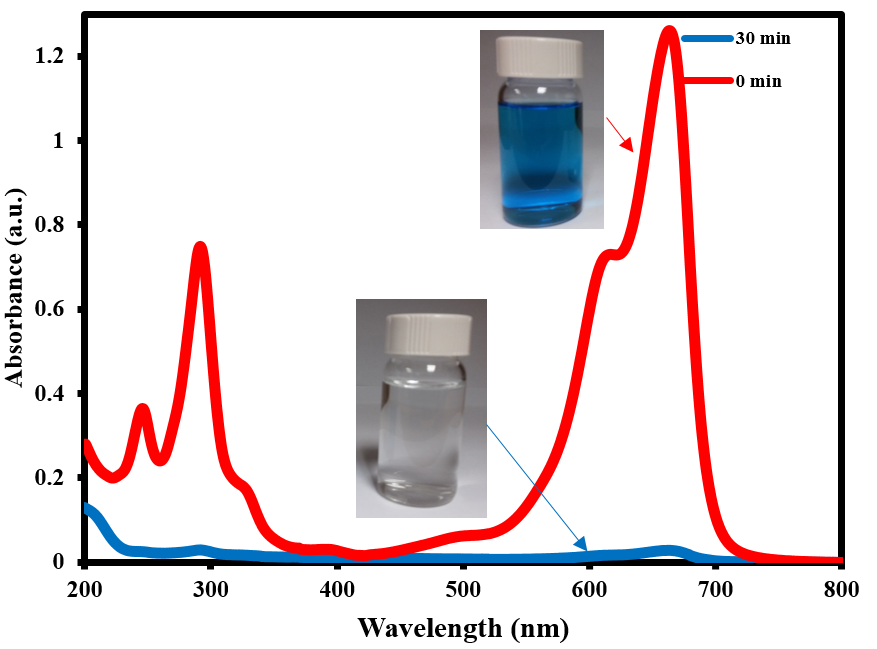

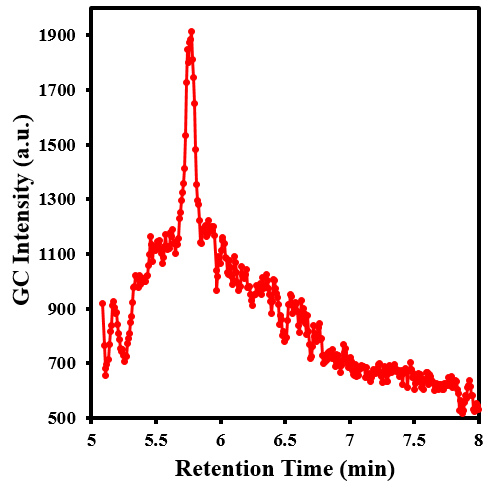

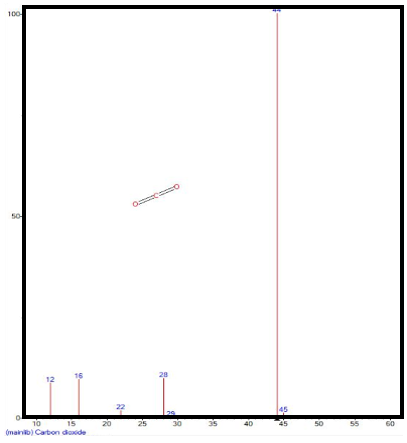


**(c)**

**(b)**

**(a)**

**S1 Fig.** (a) UV-Vis spectra of MB solution in the presence of BiOCl-24 under visible light irradiation showing that no absorption was noticed for lower hydrocarbon products was noticed, (b) GC chromatogram of CO_2_ showing presence of CO_2_ peak in the degraded product and (c) Mass spectrum of CO_2_.
